# Altered expression of Toll-like receptor 9 in the lung tissue of adult mice generated by in vitro embryo culture and embryo transfer

**DOI:** 10.1007/s00418-026-02498-2

**Published:** 2026-07-02

**Authors:** Murat Öztürk, Göksel Doğan, Levent Karagenç

**Affiliations:** Department of Histology-Embryology, Faculty of Veterinary Medicine, Aydın Adnan Menderes University, 09000 Aydın, Türkiye

**Keywords:** ART, In vitro embryo culture and embryo transfer, Lung tissue, Mouse, Toll-like receptor 9

## Abstract

Children conceived through assisted reproductive technologies (ART) are more sensitive to asthma and allergic respiratory diseases compared with children born through natural pregnancy. Growing evidence suggests that Toll-like receptors (TLRs) play a role in asthma. We previously demonstrated that TLR9 expression is significantly downregulated in the lung tissue of mouse fetuses generated through ART. The present study therefore investigated whether altered expression of TLR9 is maintained in the lung tissue in the perinatal period and in adult mice conceived through in vitro embryo culture and embryo transfer. We also examined the expression pattern of TLR9 in lung tissue. The study comprised one control (CG) and one experimental group (EG). Mice comprising EG were generated through the transfer of in vitro-derived F2 blastocysts. Mice obtained from naturally ovulated females served as the CG. Quantitative real-time PCR (qRT-PCR) and immunohistochemistry/immunofluorescence analyses were employed to determine the expression of TLR9. qRT-PCR analyses demonstrated that expression of TLR9 decreased 1.38-fold in EG when compared with the CG. Immunohistochemistry/immunofluorescence analyses revealed that the expression pattern of TLR9 is similar between the groups. Simultaneous staining of sequential sections further elucidated that TLR9 is expressed in type II alveolar epithelial cells. It therefore appears that altered expression of TLR9 observed in the lung tissue of mouse fetuses generated through embryo culture and embryo transfer persists into adulthood. To what extent the findings of the present study apply to human ART warrants further investigations.

**Supplementary Information:**

The online version contains supplementary material available at 10.1007/s00418-026-02498-2.

## Introduction

Infertility is defined as a failure to achieve a clinical pregnancy after 12 months or more of regular unprotected sexual intercourse (Zegers-Hochschild et al. 2009). Infertility, recognized as a global public health issue by the World Health Organization (WHO), affects approximately 17.5% of couples of reproductive ages worldwide (WHO 2023). Today, various assisted reproductive technologies (ART) are used in the treatment of infertility. Of these, in vitro embryo culture and embryo transfer are among the most commonly employed procedures. Most recent estimates indicate that the number of babies born through ART exceeds ten million (Pinborg et al. 2023; ESHRE 2025). Nevertheless, there remain legitimate concerns over the safety of ART due to a large body of evidence suggesting that children born through ART may be at a higher risk for various health problems (Schieve et al. 2002; Jackson et al. 2004; Klemetti et al. 2006; McDonald et al. 2009; Tomic and Tomic 2011; Pandey et al. 2012; Qin et al. 2017) including asthma (Ericson et al. 2002; Carson et al. 2011, 2013; Halliday et al. 2019; Kuiper et al. 2019; Wijs et al. 2022; Liu et al. 2024) compared with children conceived naturally. Asthma, characterized by inflammation and narrowing of the small airways in the lung tissue (Porsbjerg et al. 2023), is a heterogenous disease affecting over 330 million individuals worldwide (Sockrider and Fussner 2020; Papadopoulos et al. 2022). The pathophysiology of asthma is a highly complex and multifactorial process (Maddox and Schwartz 2002) involving several different inflammatory cells and multiple mediators (Xu et al. 2025). There is emerging evidence indicating that TLR9 might be involved in the pathobiology of asthma (Murakami et al. 2020; Zhao et al. 2020).

TLR9 recognizes DNA containing CpG motifs derived from bacteria and viruses (Hoffjan et al. 2005). It is well established that the expression of TLR9 has been observed in bronchial epithelium, vascular endothelium, alveolar septal cells, and alveolar macrophages in both mouse and human lung tissue (Schneberger et al. 2013). Furthermore, the expression of TLR9 by type II alveolar epithelial cells in mouse lungs was demonstrated by immuno-electron microscopy (Schneberger et al. 2013). Type II alveolar epithelial cells are increasingly recognized as key regulators of airway immune homeostasis as they play a central role in host defense by maintaining a robust alveolar epithelial barrier, coordinating innate immune responses, and secreting surfactant proteins (Ruaro et al. 2021). It is interesting to note that the expression of Sp-C (a specific marker of type II alveolar epithelial cells) is altered in the lung tissue of mice generated by in vitro embryo culture and embryo transfer (Doğan et al. 2024a). Although underlying mechanisms remain obscure, evidence demonstrating that TLR9 might be involved in the pathobiology of asthma (Murakami et al. 2020; Zhao CC et al. 2020) raises the possibility that altered expression of TLR9 might be involved in this process. Our previous findings indicating that the expression of transcripts encoding TLR9 is significantly downregulated in the lung tissue of mouse fetuses generated through embryo culture and embryo transfer (Doğan et al. 2024b) lend support to this hypothesis.

There is accumulating data indicating that TLR9 expression is highly responsive to epigenetic alterations (Fernández et al. 2022; Wang 2025). Whether culture of embryos under suboptimal conditions, as is the case in the present study, alters the expression of TLR9 through epigenetic modulations remains to be determined as demonstrated previously for several genes including H19 (Wang et al. 2012; Smith et al. 2015; Hattori et al. 2019), Igf2 (Wang et al. 2012; Smith et al. 2015; Hattori et al. 2019), Kcnq1ot1 (Wang et al. 2012), Igf2r (Smith et al. 2015), Snrpn (Smith et al. 2015), and Cdkn1c (Wang et al. 2012). Answering these questions is a crucial step in establishing a mechanistic explanation underlying how ART-related stress imposed at preimplantation stages of development through ovarian stimulation, in vitro embryo culture, and/or embryo transfer modulates innate immune function in the offspring and renders them more susceptible to various diseases including asthma later in life (Ericson et al. 2002; Carson et al. 2011, 2013; Halliday et al. 2019; Kuiper et al. 2019; Wijs et al. 2022; Liu et al. 2024). As a prelude to addressing these questions, it is essential to first determine whether the reduced expression of TLR9 observed in the lung tissue of mouse fetuses generated through embryo culture and embryo transfer persists into adulthood. We therefore aimed to investigate whether the expression of TLR9 is altered in the lung tissue of adult mice conceived through in vitro embryo culture and embryo transfer. We also examined the expression pattern of TLR9 in ART-generated mice using immunohistochemistry and immunofluorescence.

## Materials and methods

### Animals

Zygotes, embryos, and adult mice were obtained from 8- to 10-week-old female F1 hybrid (C57BL/6 × BALBc) mice. Mice were maintained on a 14-h light/10-h dark photoperiod. Food and water were supplied ad libitum. The experiment comprised one experimental (EG) and one control (CG) group. F2 adult mice (8 weeks old) comprising the EG (*n* = 15) were generated through the transfer of in vitro-derived day 5 F2 blastocysts to pseudo-pregnant female mice (see details below). F2 adult mice (8 weeks old) obtained from naturally ovulating females served as the CG (*n* = 15). Adult lung tissue samples of male mice generated through the transfer of in vitro-derived F2 blastocysts (EG) and F2 adult mice (CG) were used in the present study (Doğan et al. 2024a). Expression of TLR9 in the lung tissue was analyzed using qRT-PCR, immunohistochemistry, and immunofluorescence.

### Generation of adult mice comprising the experimental group (EG)

In order to generate adult mice for the EG, multiple ovulations were induced by 5 IU pregnant mare’s serum gonadotrophin (Folligon, Intervet, Türkiye) followed 48 h later by 5 IU hCG (Pregnyl; Organon, Türkiye). F1 females were placed with F1 males following the administration of hCG, and the presence of the vaginal plug was checked the following morning. Cumulus-enclosed eggs were collected from super-ovulated mice at 22 h post-hCG in Quinn’s Advantage medium supplemented with HEPES (QA-HEPES, Cooper Surgical, Trumbull, CT, USA) and 5 mg/ml protein (LifeGlobal® Protein Supplement, Cooper Surgical, Trumbull, CT, USA). Cumulus cells were removed by incubating the eggs in medium QA-HEPES/LifeGlobal® Protein Supplement containing hyaluronidase (0.5 mg/ml; Sigma Chemical Co., St Louis, MO, USA) for a short period of time. Embryos were cultured as described by Karagenc et al. (2004, 2005; Karagenç et al. 2019). Briefly, zygotes were washed first in three large drops of QA-HEPES/LifeGlobal® Protein Supplement and then in Quinn’s Advantage Cleavage medium (Cooper Surgical, Trumbull, CT, USA) supplemented with 5 mg/ml LifeGlobal® Protein Supplement (QA-Cleavage/LifeGlobal® Protein Supplement). In all experiments, embryos were cultured in 20 μl droplets of pre-equilibrated QA-Cleavage/LifeGlobal® Protein Supplement in groups of ten under embryo-tested paraffin oil (LifeGlobal, Cooper Surgical, Trumbull, CT, USA) for 46 h to around 8-cell/compaction stage. After 46 h of culture, embryos were washed in QA-Blastocyst medium (QA-Blastocyst, Cooper Surgical, Trumbull, CT, USA) supplemented with 5 mg/ml of LifeGlobal® Protein Supplement and cultured in 20 μl droplets of pre-equilibrated QA-Blastocyst/LifeGlobal® Protein Supplement for an additional 49 h to the blastocyst stage. In vitro embryo culture was performed under atmospheric concentrations of oxygen for the entire culture period (95 h exposure to 20% oxygen). In all in vitro culture experiments, embryo development was assessed on day 3 at 9 a.m. (46 h culture), on day 4 at 4 p.m. (78 h culture), and on day 5 at 9 a.m. (95 h culture) as described previously (Gardner and Lane 2004; Karagenc et al. 2004). In vitro culture experiments were repeated three times. Zygotes grown to the blastocyst stage were transferred to day 4 pseudo-pregnant F1 female mice (− 1 day asynchronous) to generate F2 adult mice comprising the EG group. To this end, expanded and hatching blastocysts were randomly allocated to each uterine horn, and five blastocysts were transferred per uterine horn.

### qRT-PCR

RNA extraction, cDNA synthesis, and qRT-PCR were performed as described by Karagenç et al. (2019). qRT-PCR analyses were performed using three independent RNA pools. cDNA was synthesized using 500 ng of total RNA using the HighCapacity cDNA Reverse Transcription Kit (Applied Biosystems, Waltham, MA, USA) and random primers as described by the manufacturer. In order to determine the expression of TLR9, TaqMan Gene Expression Assay (Applied Biosystems, Foster City, CA, USA) for murine TLR- (Tlr-9; Mm0044446193-M1) was used. Murine* Actb* (β-actin, Mm00607939_s1) and* Gapdh* (Gapdh; Mm99999915-G1) were used as endogenous reference genes. qRT-PCR were performed in a reaction volume of 20 µl, comprising 1 µl of assay probes (β-actin or TLR9), 10 µl of TaqMan® Universal PCR Master Mix, 7 µl of nuclease-free water, and 2 µl of cDNA samples. Reactions were performed in triplicate on an Applied Biosystems StepOne thermal cycler. Reaction conditions were pre-optimized, beginning with a 2 min incubation at 50 °C, followed by enzyme activation at 95 °C for 10 min. This was succeeded by forty cycles of denaturation at 95 °C for 15 s and annealing/elongation at 60 °C for 60 s. Results were normalized to the endogenous reference gene (β-actin), and the difference in expression relative to the control group was calculated using the 2^−ΔΔCt^ method (Schmittgen and Livak 2008).

### Immunohistochemistry and immunofluorescence

Lung tissue samples were fixed in 4% paraformaldehyde (pH 7.4) at 4 °C overnight, dehydrated through a graded series of ethanol and embedded in Paraplast X-TRA (Leica, Wetzlar, Germany). Immunohistochemistry was employed to characterize the immunopositivity of cells expressing TLR9 using anti-TLR9 (NBP2-24,729, 1:100; Novus Biotechnology, Centennial, CO, USA) as previously detailed by Doğan et al. (2024a, b). To further demonstrate the expression of TLR9 by type II alveolar epithelial cells, sequential sections taken at 5 µm intervals were stained using an antibody specific for type II alveolar epithelial cells. To this end, thin (5 μm) sections were bleached in 3% H_2_O_2_ for 10 min, washed in Tris buffer solution (TBS, pH 7.6) three times for 5 min each, and blocked in blocking solution (Histostain Plus Broad Spectrum, Invitrogen, Carlsbad, CA, USA) for 20 min. Sections were incubated with anti-SpC (a specific marker of type II alveolar epithelial cells, FL-197, sc-13970, 1:100, Santa Cruz Biotechnology, Dallas, TX, USA) overnight at 4 °C. Sections were rinsed once in TBS and then washed 3 × 5 min in TBS. Sections were then incubated in biotinylated secondary antibody (Histostain Plus Broad Spectrum, Invitrogen) at 37 °C for 1 h, rinsed/washed in TBS, and then incubated in streptavidin HRP (Histostain Plus Broad Spectrum, Invitrogen, Carlsbad, CA, USA) for 1 h at 37 °C. After a final rinse and wash in TBS, immunopositive cells were detected using 3,3′-diaminobenzidine tetrahydrochloride (DAB, Sigma-Aldrich, St. Louis, MO, USA) solution (3 mg/mL in Tris–HCl, pH 7.6, with 3% H_2_O_2_). Harris hematoxylin was used for nuclear counterstaining. Immunofluorescence staining was performed to characterize the immunopositivity of cells expressing TLR9. To this end, sections were blocked in a blocking solution comprising 5% normal goat serum supplemented with 0.1% Triton X/TBS for 1 h at room temperature. Sections were then incubated with anti-TLR9 (NBP2-24,729, 1:100; Novus Biotechnology, Centennial, CO, USA) overnight at 4 °C. Following a 3 × 5 min wash in TBS, sections were incubated with an Alexa Fluor 555 secondary antibody (Invitrogen, A21127, Carlsbad, CA, USA) at 37 °C for 1 h. Cell nuclei were counterstained with DAPI (4′,6-diamidino-2-phenylindole, Invitrogen, Carlsbad, CA, USA). The specificity of primary antibodies was validated using several appropriate negative controls: omission and replacement of primary antibodies with TBS (negative reagent control); omission and replacement of primary antibodies with normal mouse serum for TLR9 and normal rabbit serum for SpC (blocking controls); omission and replacement of monoclonal primary antibody against TLR9 with an irrelevant mouse IgG1 monoclonal antibody against anti-GFAP (isotype control, 2-A5, sc-65343, 1:100, Santa Cruz Biotechnology, Dallas, TX, USA). Tracheal tissue known to lack type II alveolar epithelial cell markers was also used as a negative tissue control for anti-SpC rabbit polyclonal primary antibody. Sections were observed on an Olympus BX51 light microscope (Olympus, Tokyo, Japan), equipped with UPlanApo ×20 (NA 0.75), ×40 (NA 0.90), and ×100 (NA 1.40) oil objectives. Fluorescent images were obtained using the same microscope equipped with a fluorescence attachment using the following filter cubes: Olympus filter cube U-MWIG3 (excitation filter 530–550; emission filter 575IF); Olympus filter cube U-MWU2 (excitation filter 330–385; emission filter 420). Images were captured using Olympus DP70 camera with DP controller software (Ver. 3.1.1.267) under identical detector gain, exposure settings, and spatial resolution, using consistent acquisition parameters across all images.

### Statistical analysis

Shapiro–Wilk test was used to assess the normality of the values. Analyses of parameters passing the normality test were performed using unpaired* t* test. Data comprising CG and EG were shown as mean ± standard deviation. The statistical package SPSS (IBM SPSS Statistics for Windows, Version 21.0. Armonk, NY: IBM Corp.) was used in all statistical analyses. The value of *p* < 0.05 was considered significantly different.

## Results

### Expression of TLR9 in adult lung tissue

**Fig. 1:**
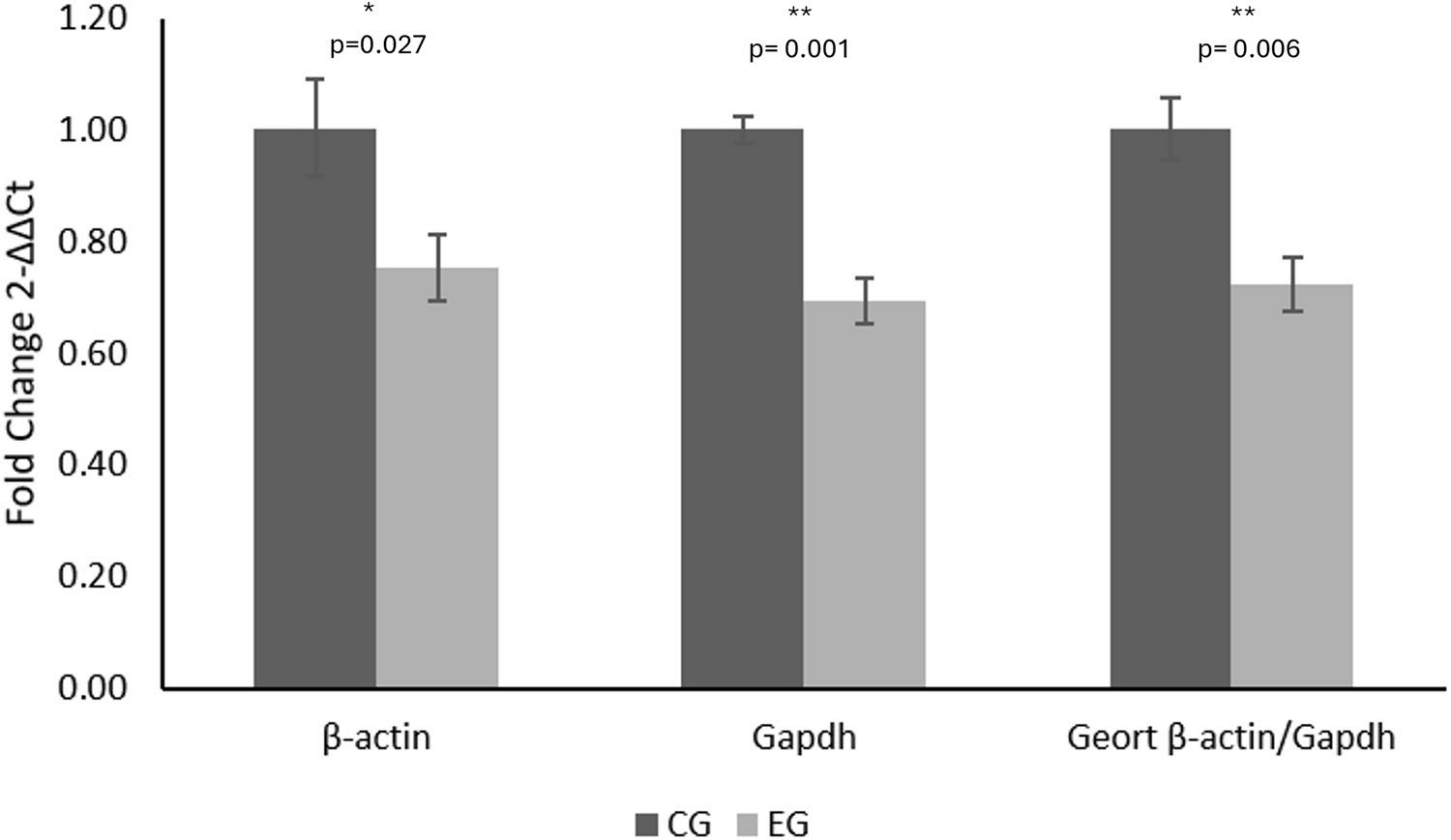
Real-time PCR analysis of TLR9 in lung tissues of CG and EG. Transcript levels were quantified using TaqMan qRT-PCR. Beta-actin, Gapdh, and geometric means of the expression of the two endogenous reference genes were used for normalization of Ct values obtained for TLR9 gene. Fold changes in gene expression were calculated using 2^−ΔΔCt^ method. *p < 0.05; **p < 0.01

qRT-PCR analyses demonstrated that mRNA levels for TLR9 were significantly reduced in EG using both β-actin (relative fold change − 1.33 ± 0.09, *p* = 0.027) and GAPDH (relative fold change − 1.45 ± 0.03, *p* = 0.001) as endogenous control genes. Similar results were obtained when geometric means of the expression of the β-actin and GAPDH was used (a relative fold change of − 1.38 ± 0.06, *p* = 0.006; Fig. 1).

### Expression pattern of TLR9 in adult lung tissue

**Fig. 2:**
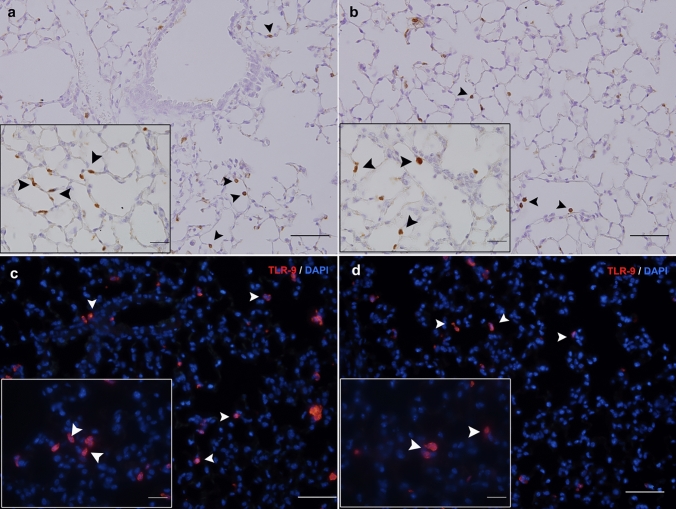
Expression of TLR9 in the adult lung tissue of CG and EG mice. Immunohistochemistry and immunofluorescence were used to determine cells expressing TLR9. Harris hematoxylin and DAPI were used as a counterstain for cell nuclei. Cells expressing TLR9 appear brown in color (**a**, **b**, arrowheads) using immunohistochemistry in the lung tissues of CG (**a**) and EG (**b**). Red fluorescence staining of cells expressing TLR9 (**c**, **d**, arrowheads) was observed in the lung tissue of both CG (**c**) and EG (**d**). Please note that immunopositive cells in inset boxes were visualized using a ×100 objective. Scale bars are 50 µm (**a**–**d**) and 20 µm (inset boxes)

**Fig. 3:**
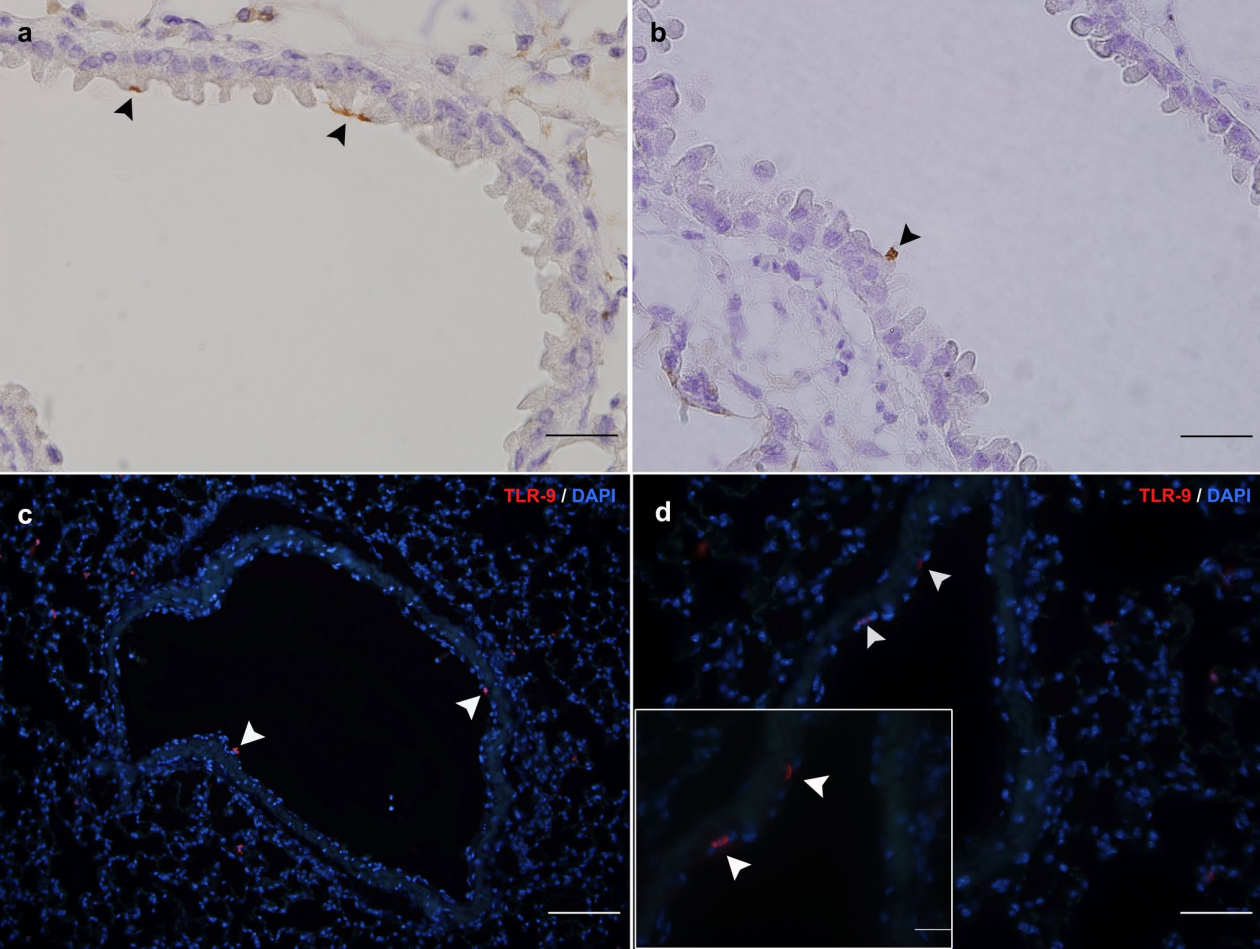
Expression of TLR9 in the airway epithelial cells in adult lung tissue of CG and EG mice. Immunohistochemistry and immunofluorescence were used to determine cells expressing TLR9. Harris hematoxylin and DAPI were used as a counterstain for cell nuclei. Cells expressing TLR9 appear brown in color (**a**, **b**, arrowheads) using immunohistochemistry in the lung tissues of CG (**a**) and EG (**b**). Red fluorescence staining of cells expressing TLR9 (**c**, **d**, arrowheads) was observed in the lung tissue of CG (**c**) and EG (**d**). Please note that TLR9 was expressed in the airway epithelial cells (**a**–**d**). Please note that immunopositive cells in inset boxes were visualized using a ×100 objective. Scale bars are 100 µm (**c**), 50 µm (**d**), and 20 µm (**a**, **b**, inset boxes)

**Fig. 4:**
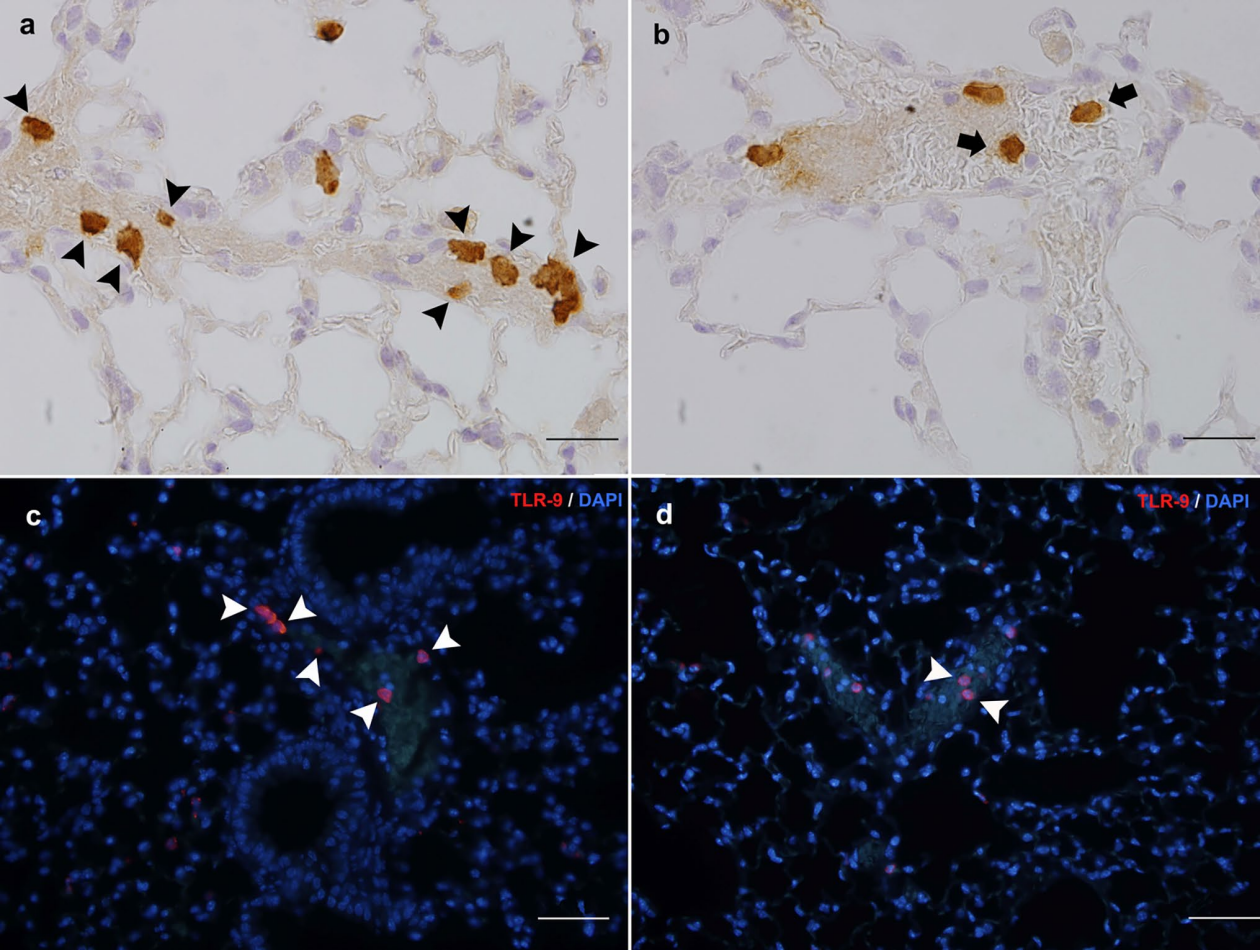
Expression of TLR9 in the vascular lumen in adult lung tissue of CG and EG mice. Immunohistochemistry and immunofluorescence were used to determine cells expressing TLR9. Harris hematoxylin and DAPI were used as a counterstain for cell nuclei. Cells expressing TLR9 appear brown in color (**a**, **b**, arrowheads) using immunohistochemistry in the lung tissues of CG (**a**) and EG (**b**). Red fluorescence staining of cells expressing TLR9 (**c**, **d**, arrowheads) was observed in the lung tissue of CG (**c**) and EG (**d**). Please note that TLR9 was expressed in the cells within vascular lumen (**a**–**d**, arrowheads). Scale bars are 50 µm (**c**, **d**) and 20 µm (**a**, **b**)

**Fig. 5:**
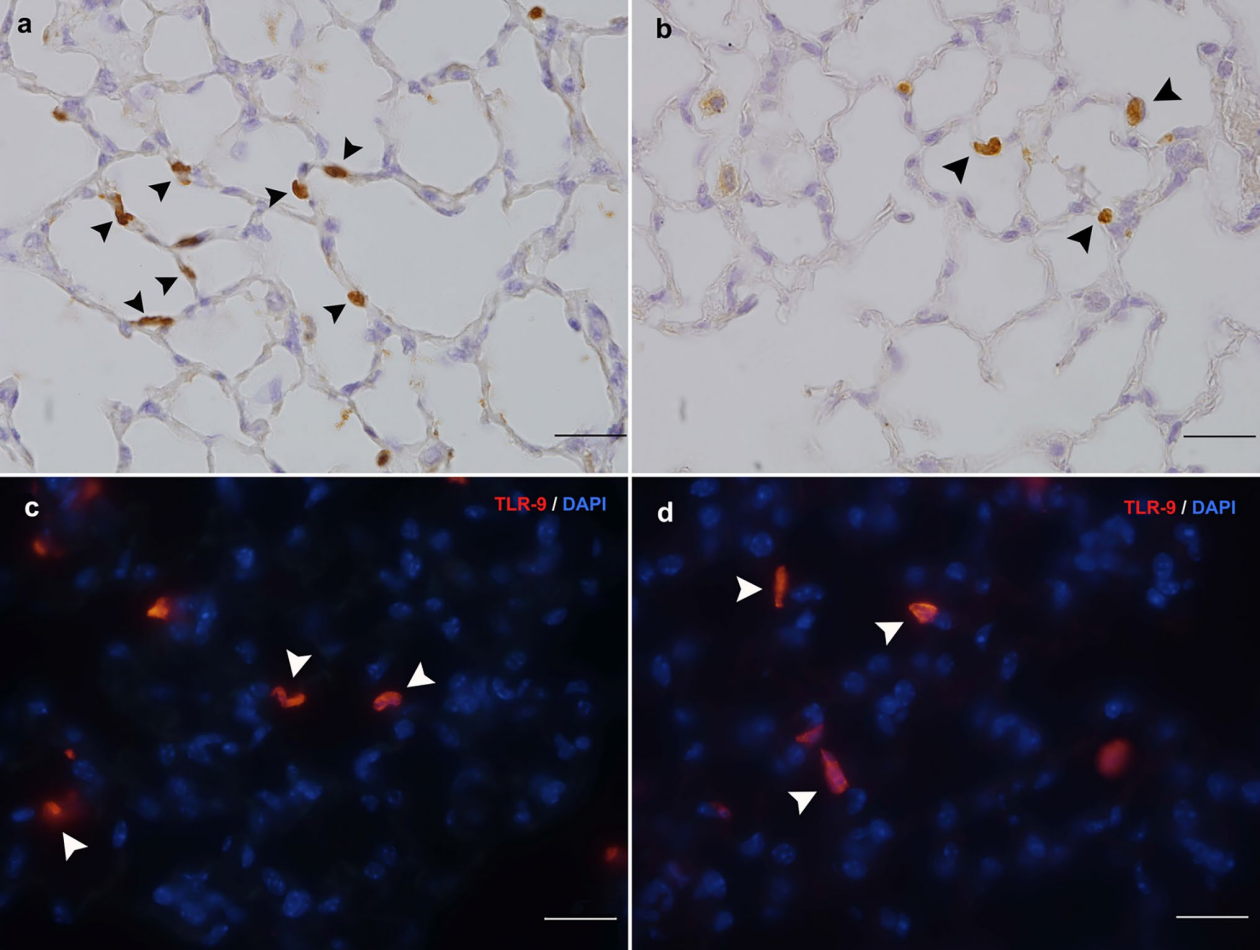
Expression of TLR9 in the alveolar walls in adult lung tissue of CG and EG mice. Immunohistochemistry and immunofluorescence were used to determine cells expressing TLR9. Harris hematoxylin and DAPI were used as a counterstain for cell nuclei. Cells expressing TLR9 appear brown in color (**a**, **b**, arrowheads) using immunohistochemistry in the lung tissues of CG (**a**) and EG (**b**). Red fluorescence staining of cells expressing TLR9 (**c**, **d**, arrowheads) was observed in the lung tissue of CG (**c**) and EG (**d**). Please note that TLR9 was expressed in the alveolar walls (**a**–**d**). Scale bars are 20 µm

**Fig. 6:**
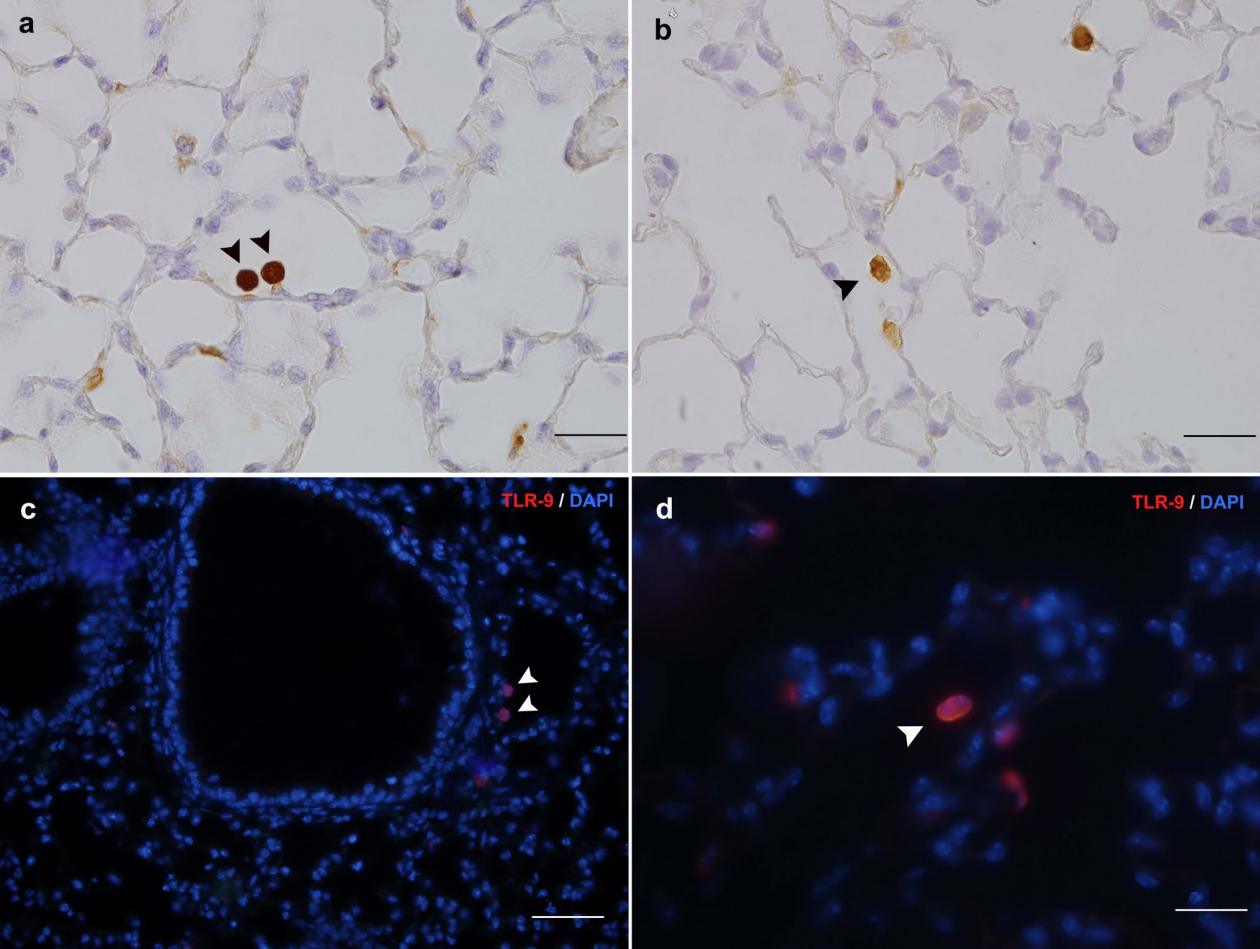
Expression of TLR9 in the alveolar lumen in adult lung tissue of CG and EG mice. Immunohistochemistry and immunofluorescence were used to determine cells expressing TLR9. Harris hematoxylin and DAPI were used as a counterstain for cell nuclei. Cells expressing TLR9 appear brown in color (**a**, **b**, arrowheads) using immunohistochemistry in the lung tissues of CG (**a**) and EG (**b**). Red fluorescence staining of cells expressing TLR9 (**c**, **d**, arrowheads) was observed in the lung tissue of CG (**c**) and EG (**d**). Please note that TLR9 was expressed in the alveolar lumen (**a**–**d**). Scale bars are 50 µm (**c**) and 20 µm (**a**, **b**, **d**)

In light of evidence demonstrating that TLR9 is expressed in different anatomical regions in the mouse lung tissue (Schneberger et al. 2013), we determined the expression pattern of TLR9 in adult lung tissue samples using immunohistochemistry and immunofluorescence. Immunohistochemical and immunofluorescence analyses revealed a similar pattern of expression in both groups in terms of staining intensity, localization, and overall morphology (Fig. 2). TLR9 expression was detected in airway epithelial cells (Fig. 3), in cells in the vascular lumen (Fig. 4), in alveolar walls (Fig. 5), and alveolar lumen (Fig. 6). No immunopositivity was observed in negative control sections (Supplementary Figs. 1–4).

**Fig. 7:**
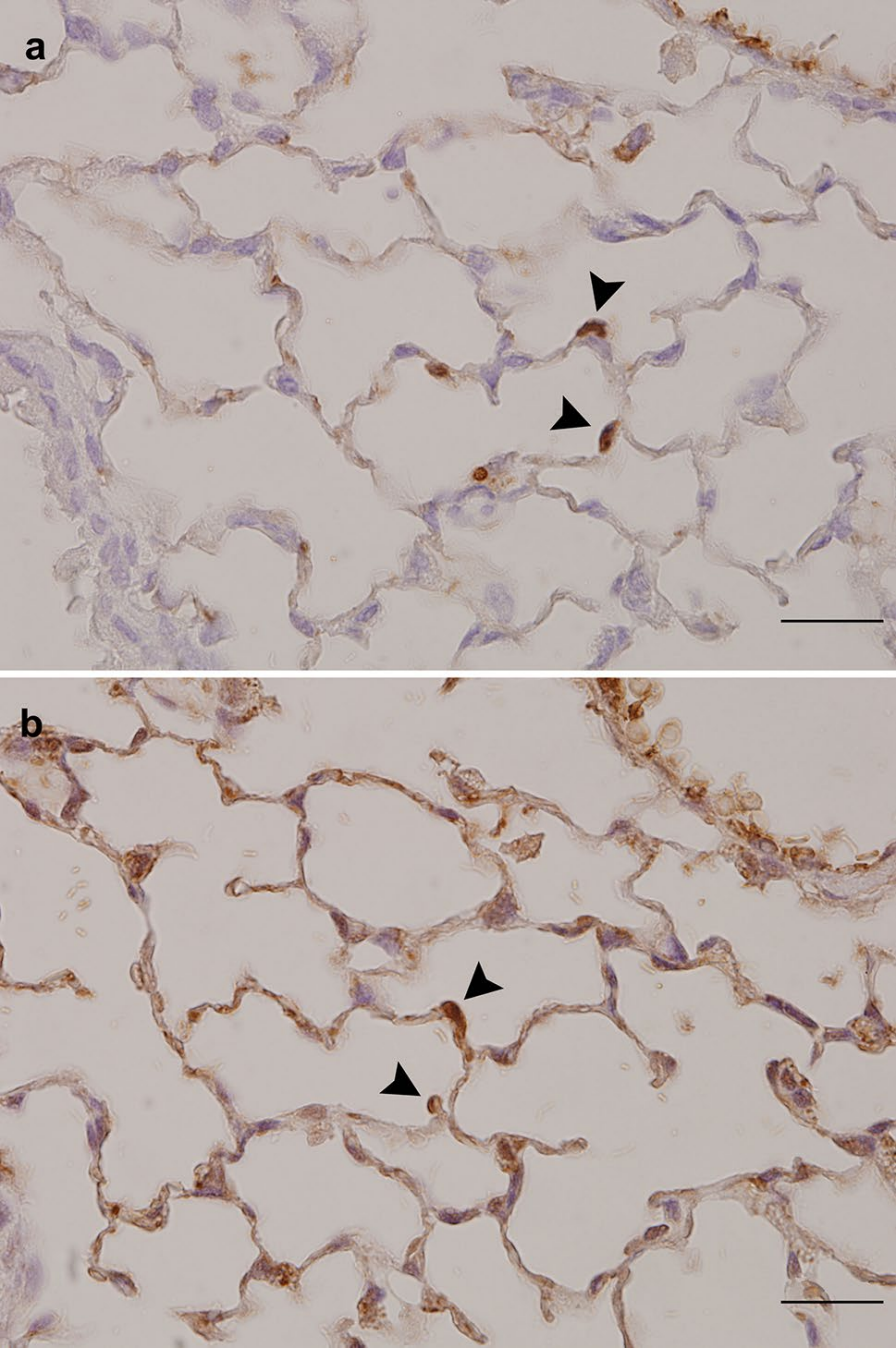
Expression of TLR9 by type II alveolar epithelial cells in the adult lung tissue of CG mice. Immunohistochemistry was conducted on sequential sections taken at 5 µm intervals. The pictures in **a** and **b** are mirror images with at 5 µm interval. Harris hematoxylin was used for nuclear counterstaining. Sequential sections (**a**, **b**) were stained using anti-TLR9 (**a**) along with anti-Sp-C (a specific marker for type II alveolar epithelial cells, **b**). Immunopositive cells expressing both Sp-C (**b**) and TLR9 (**a**) appear brown in color (arrowheads). Please note that type II alveolar epithelial cells expressing Sp-C (**b**, arrow heads) are also immune positive for TLR9 (**a**, arrowheads). Scale bars are 20 µm

Having determined that TLR9 expression pattern is similar between CG and EG in the lung tissue, we wanted to determine if TLR9 is expressed by type II alveolar epithelial cells. To this end, sequential sections taken at 5 µm intervals were stained simultaneously using anti-TLR9 antibody along with anti-Sp-C. The results indicate that TLR9 is expressed by type II alveolar epithelial cells (Fig. 7).

## Discussion

Since the birth of Louise Brown, the first baby conceived through in vitro fertilization and embryo transfer (Edwards and Steptoe 1978), more than ten million babies have been estimated to be born worldwide through ART (Pinborg et al. 2023; ESHRE 2025). Various ART procedures including intracytoplasmic sperm injection (ICSI), in vitro fertilization, in vitro embryo culture and embryo transfer constitute an indispensable component of current infertility treatments. Nevertheless, there remain legitimate safety concerns, supported by extensive evidence suggesting that children born through ART may have an increased likelihood of health problems, including asthma and allergic respiratory diseases, when compared with naturally conceived children (Zuppa et al. 2001; Koivurova et al. 2003; Jackson et al. 2004; Bonduelle et al. 2005; Klemetti et al. 2006; Knoester et al. 2008; McDonald et al. 2009; Pandey et al. 2012; Hansen et al. 2013; Qin et al. 2017; Zhao et al. 2020b; Mitter et al. 2022; Pettersson et al. 2022). A relatively high incidence of very low birth weight (approx. 3–6%, Sunderam et al. 2022; Faa et al. 2024), low birth weight (approx. 18–32%, Sunderam et al. 2019, 2022; Faa et al. 2024), and small for gestational age (approx. 3–13%, Qu et al. 2020; Gorgui et al. 2022; Singh et al. 2024) have been reported in ART pregnancies, frequently in association with fetal growth restriction. These outcomes are of particular clinical significance, as they are recognized as major risk factors for the development of asthma (Ericson et al. 2002; Carson et al. 2011, 2013; Halliday et al. 2019; Kuiper et al. 2019; Wijs et al. 2022) and impaired lung function (Dharmage et al. 2019; Yang et al. 2020) in adulthood. Nevertheless, to what extent and how ART renders individuals more susceptible to asthma remains obscure.

Asthma is a heterogeneous disease comprising multiple phenotypes such as allergic asthma, non-allergic asthma, late-onset asthma, asthma with fixed airflow limitation, and obesity-associated asthma (Wenzel 2012; Xu et al. 2025). Respiratory symptoms of asthma including dyspnea, cough, wheezing, and chest tightness, are key components in the diagnosis of the disease and are widely used to assess treatment response and overall disease control (Krishnan et al. 2012). Asthma not only contributes to increased morbidity (Valet et al. 2011; Centers for Disease Control and Prevention (CDC), 2011) but also impacts fertility (Virchow 2012; Gade et al. 2014; Juul Gade et al. 2014; Bláfoss et al. 2019; Jöud et al. 2022; Bravo-Solarte et al. 2023; Shaikh et al. 2023) and early embryonic development (Wafriy et al. 2021; 2023). In fact, women with asthma are more likely to seek ART (Vejen Hansen et al. 2019; Jöud et al. 2022). Allergic asthma may also impair preimplantation embryo development (Wafriy et al. 2021; 2023). Maternal asthma, affecting nearly 20% of all pregnancies (Das et al. 2022), is associated with an increased risk of neonatal respiratory morbidities, including transient tachypnea of the newborn (TTN), respiratory distress syndrome (RDS), and reduced lung function at 5–6 weeks of age (Murphy et al. 2013; Mendola et al. 2014; de Gouveia Belinelo et al. 2021). Children born to asthmatic mothers are more likely to develop asthma, wheezing, pneumonia, upper respiratory tract infections, and general respiratory morbidity (Lim et al. 2010; Spiegel et al. 2018; Venter et al. 2021). Furthermore, maternal asthma is associated with impaired lung function and an elevated risk of asthma later in life (Lim et al. 2010; Damgaard et al. 2015; Berry et al. 2016). Data gathered from a mouse model confirm these findings and further demonstrate that the process involves epigenetic changes associated with increased expression of genes related to glucocorticoid signaling in the lung centered on group 2 innate lymphoid cells during fetal life (Takao et al. 2025).

There is emerging evidence suggesting that members of the TLR family including TLR9 examined in the present study play pivotal functions in the pathobiology of asthma (Xu et al. 2025) by modulating the TLR9–interleukin (IL)-2 axis, which play a key role in Th2-driven inflammation by regulating IL-17A production in allergic asthma (Murakami et al. 2020). Moreover, TLR9 was shown to drive NLRP3 inflammasome activation and oxidative stress in murine allergic airway inflammation (Zhao et al. 2020). We previously demonstrated that preimplantation stress caused by atmospheric oxygen, even for a short period of time, leads to fetal growth restriction, impaired lung development and redox balance along with dysregulation of several genes in oxidative stress response (Karagenç et al. 2019), a process accompanied by downregulation of TLR9 (Doğan et al. 2024b). We therefore aimed in the present study to determine whether altered expression of TLR9 observed in the perinatal period persists into adulthood in the lung tissue of mice conceived through in vitro embryo culture and embryo transfer.

Reduced expression of TLR9 in the lung tissue of adult mice conceived through in vitro embryo culture and embryo transfer is the most important finding of the present study. Considering our previous findings demonstrating that expression of TLRs is also altered in the lung tissue mouse fetuses (Doğan et al. 2024b), reduced expression of TLR9 observed in the present study further suggests that innate immune response mediated by lung cells including type II alveolar epithelial cells might be compromised. It is also interesting to note that TLR9 not only initiates immune responses (Bhan et al. 2007; Knuefermann et al. 2007; Suresh et al. 2016) but also regulates physiological metabolism of non-immune cells including alveolar epithelial cells. TLR9 can exacerbate pulmonary fibrosis by promoting NLRP3-mediated pyroptosis of alveolar epithelial cells (Ren et al. 2024). TLR9-mediated sensing of tissue damage can also promote an inflammatory response during early infection, driven by epithelial cells including type II alveolar epithelial cells (Kim et al. 2025). We recently demonstrated that expression of transcripts encoding Sp-c (a specific marker of type II alveolar epithelial cells) in the adult lung tissue is upregulated (Doğan et al. 2024a). However, to what extent altered expression of Sp-c is causally linked with reduced expression of TLR9 warrants further investigations. Given evidence that TLR9 may play a role in the pathophysiology of asthma by regulating immune responses, including IL-17A production (Murakami et al. 2020), NLRP3 inflammasome activation, and oxidative stress response (Zhao et al. 2020), whether reduced expression of TLR9 renders individuals born through ART more prone to asthma and allergic respiratory diseases remains to be determined.

Immunohistochemical and immunofluorescence analyses revealed a similar pattern of expression in lung tissues in both groups. TLR9 expression was detected in airway epithelial cells, in cells in the vascular lumen, in alveolar walls, and alveolar lumen. These results were consistent with previous observations (Schneberger et al 2013). To the best of our knowledge, function of TLR9 in innate/adaptive response mediated by type II alveolar epithelial cells remains obscure. However, given that type II alveolar epithelial cells actively contribute to innate and adaptive defense through TLR signaling (Armstrong et al. 2004; Mayer et al. 2007; Thorley et al. 2007, 2011), it would be of interest to determine whether TLR9 is also downregulated in type II alveolar epithelial cells in the lung tissue of adult mice conceived through in vitro embryo culture and embryo transfer. Further studies employing loss and/or gain-of-function approaches are also warranted to determine whether altered TLR9 expression exerts functional consequences in type II alveolar epithelial cells in the adult lung tissue of ART-derived mice.

The current study demonstrated that reduced expression of TLR9 observed in the lung tissue of mouse fetuses generated through embryo culture and embryo transfer persists into adulthood and as such provides a valuable mouse model to establish a mechanistic link underlying how ART-related stress imposed at preimplantation stages of development through ovarian stimulation, in vitro embryo culture, and/or embryo transfer modulates innate immune function in the offspring and renders them more susceptible to asthma later in life. In light of evidence revealing that TLR9 expression is highly responsive to epigenetic regulations (Fernández et al. 2022; Wang 2025), it is imperative to demonstrate if epigenetic modulations are also involved in altered expression of TLR9 observed in mice generated through embryo culture and embryo transfer through longitudinal and transgenerational studies.

## Conclusion

Taken together evidence provided in the present study indicates that the expression of TLR9 is significantly downregulated in the lung tissue of adult mice conceived through in vitro embryo culture and embryo transfer, when compared to mice obtained from naturally ovulated females. It appears that altered expression of TLR9 observed in the lung tissue of mouse fetuses generated through embryo culture and embryo transfer persists into adulthood. To what extent the findings of the present study apply to human ART and how reduced expression of TLR9 contributes to asthma remain to be determined. Future longitudinal and transgenerational studies combining genetic, epigenetic, and developmental approaches should be performed to better understand how ART-related stress imposed at preimplantation stages of development renders individuals more susceptible to asthma later in life.

## Supplementary Information

Below is the link to the electronic supplementary material.

Supplementary file1 (PDF 453 KB)

## Data Availability

The data are available from the corresponding author on request.
