# Supplementary material for: Altered expression of Toll-like receptor 9 in the lung tissue of adult mice generated by in vitro embryo culture and embryo transfer
Source: Histochem Cell Biol. 2026 Jul 2;164(1):55. doi: 10.1007/s00418-026-02498-2 (PMC13328248; doi:10.1007/s00418-026-02498-2)
Supplement: Supplementary file 1 — Supplementary file1 (PDF 453 KB) [file 418_2026_2498_MOESM1_ESM.pdf]

Supplementary File

Altered expression of toll-like receptor-9 in the lung tissue of adult mice generated by in vitro embryo culture and embryo transfer

Histochemistry and Cell Biology

Murat Öztürk[1], Göksel Doğan[1], Levent Karagenç[1]*

[1]Adnan Menderes University, Faculty of Veterinary Medicine, Department of Histology-Embryology, Aydın, 09000, Turkey.

* Corresponding Author: Levent Karagenç

* Adnan Menderes University, Faculty of Veterinary Medicine, Department of Histology-Embryology, 09000, Aydın, TURKEY.

E-mail: lkaragenc@adu.edu.tr

Murat Öztürk https://orcid.org/0000-0002-4774-6980

Göksel Doğan https://orcid.org/0000-0002-4583-3140

Levent Karageç https://orcid.org/0000-0003-2074-2450

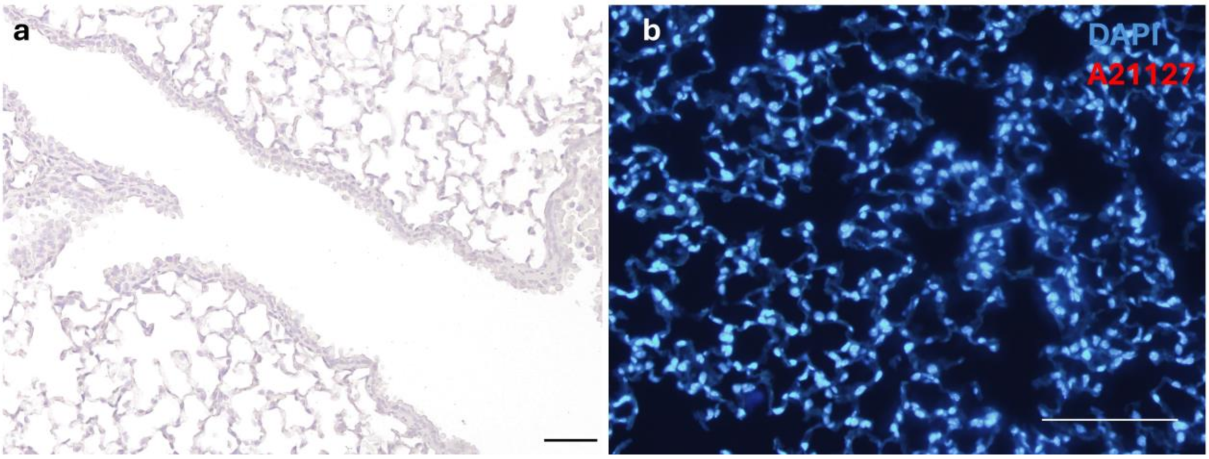

Supplementary Fig. 1. Negative Reagent Control (Omission of primary antibody) for anti-TLR-9 mouse monoclonal and anti-Sp-C rabbit polyclonal primary antibodies using immunohistochemistry and immunofluorescence. The negative control sections were treated in an identical manner except for the use of TBS (pH 7.6) instead of the anti-TLR-9 mouse monoclonal and anti-Sp-C rabbit polyclonal primary antibody. **a.** After a final rinse and wash in TBS, immune positive cells were detected using 3, 3' diaminobenzidine tetrahydrochloride (DAB) solution (3 mg/ml in Tris-HCl, pH 7,6 with 3 % H_2O_2). Sections were counterstained with Mayer's hematoxylin. **b.** Following a 3 x 5 min wash in TBS, sections were incubated with an Alexa Fluor 555 secondary antibody (Invitrogen, A21127) for anti-TLR-9 mouse monoclonal primary at 37°C for 1 h. Sections were counterstained with DAPI. The sections were observed on an Olympus BX51 microscope and images were captured using Olympus DP70 camera with DP controller software (Ver. 3.1.1.267). No immune-positivity were detected in any of the negative control sections for both TLR-9 (**a, b**) and Sp-C (**a**). Scale bars are 50 µm (**a**) and 100 µm (**b**), respectively.

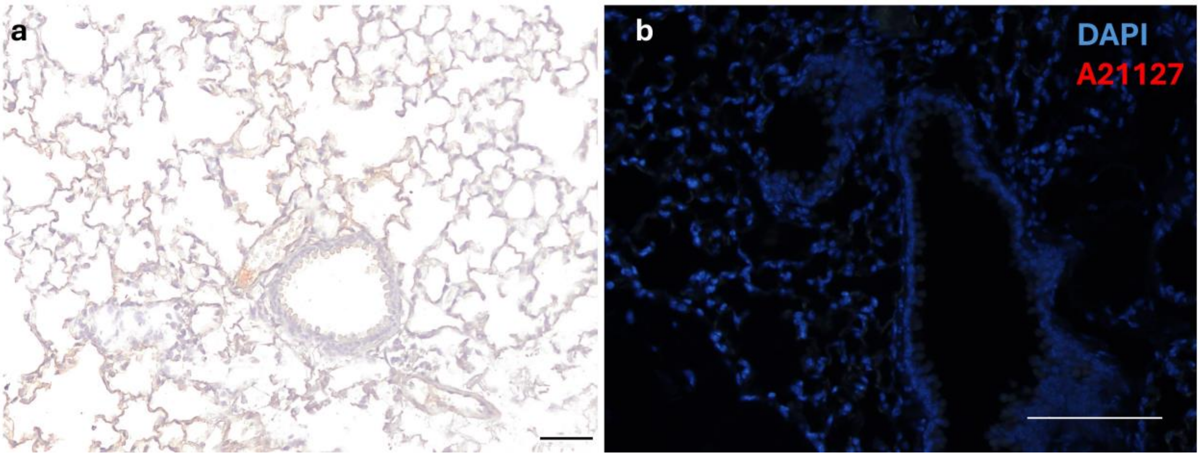

Supplementary Fig. 2. Isotype control for anti-TLR-9 mouse monoclonal primary antibody using immunohistochemistry and immunofluorescence. Sections were treated in an identical manner except for the use of anti GFAP mouse monoclonal IgG1 (1/100) instead of the anti-TLR-9 mouse monoclonal IgG1 primary antibody. **a.** After a final rinse and wash in TBS, immune positive cells were detected using 3, 3' diaminobenzidine tetrahydrochloride (DAB) solution (3 mg/ml in Tris-HCl, pH 7,6 with 3 % H_2O_2). Sections were counterstained with Mayer's hematoxylin. **b.** Following a 3 x 5 min wash in TBS, sections were incubated with an Alexa Fluor 555 secondary antibody (Invitrogen, A21127) at 37°C for 1 h. Sections were counterstained with DAPI. The sections were observed on an Olympus BX51 microscope and images were captured using Olympus DP70 camera with DP controller software (Ver. 3.1.1.267). No immune-positivity were detected in any of the isotype control sections in both immunohistochemistry (**a)** and immunofluorescence (**b**). Scale bars are 50 μm (**a**) and 100 μm (**b**), respectively.

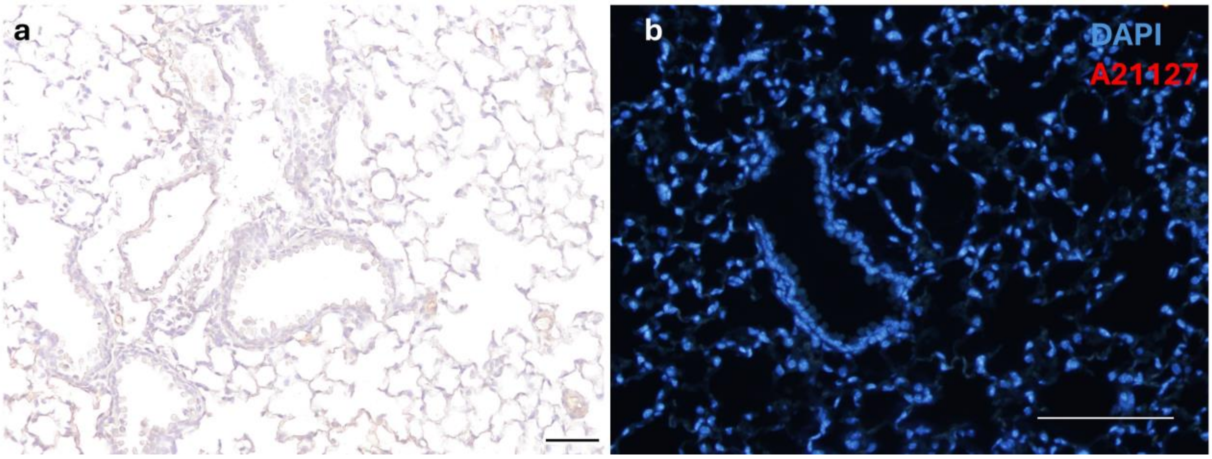

Supplementary Fig. 3. Blocking control for anti-TLR-9 mouse monoclonal primary antibody using immunohistochemistry and immunofluorescence. Sections were treated in an identical manner except for the use of normal mouse serum (1/100) instead of the anti-TLR-9 mouse monoclonal IgG1 primary antibody. **a.** After a final rinse and wash in TBS, immune positive cells were detected using 3, 3' diaminobenzidine

tetrahydrochloride (DAB) solution (3 mg/ml in Tris-HCl, pH 7,6 with 3 % H_2O_2). Sections were counterstained with Mayer's hematoxylin. **b.** Following a 3 x 5 min wash in TBS, sections were incubated with an Alexa Fluor 555 secondary antibody (Invitrogen, A21127) at 37°C for 1 h. Sections were counterstained with DAPI. The sections were observed on an Olympus BX51 microscope and images were captured using Olympus DP70 camera with DP controller software (Ver. 3.1.1.267). No immune-positivity were detected in any of the blocking control sections in both immunohistochemistry (**a**) and immunofluorescence (**b**). Scale bars are 50 µm (**a**) and 100 µm (**b**), respectively. Scale bars are 50 µm (**a**) and 100 µm (**b**).

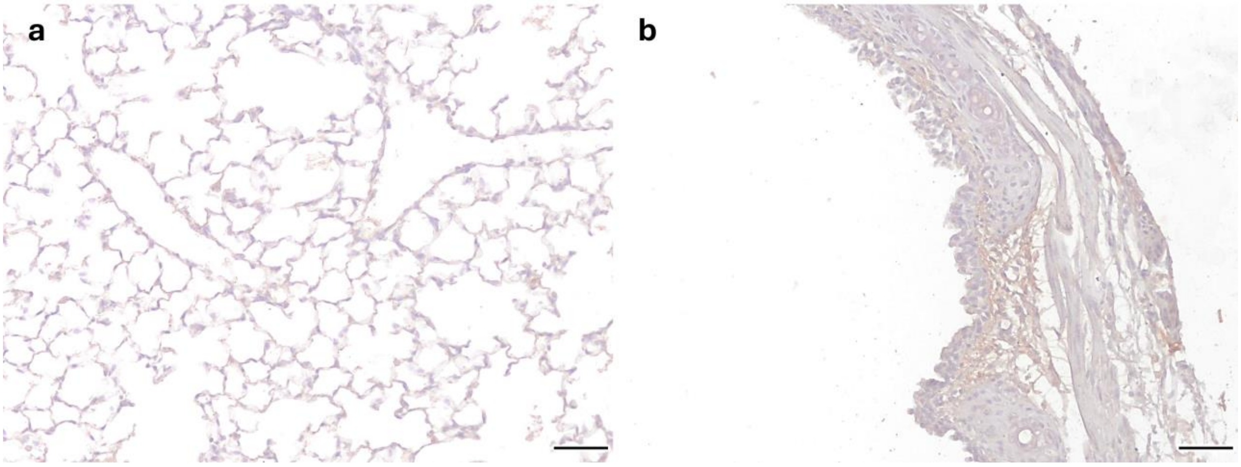

Supplementary Fig. 4. Blocking and negative tissue control for anti-Sp-C rabbit polyclonal primary antibody. The lung sections were treated in an identical manner except for the use of normal rabbit serum (1/100) instead of the anti-Sp-C rabbit polyclonal primary antibody(**a**). The tracheal tissue sections were incubated anti-Sp-C rabbit polyclonal primary antibody at 4°C for overnight (**b**). **a b,** Sections were incubated with broad spectrum second and HRP-conjugated streptavidin antibodies at 37°C for 1 h. After a final rinse and wash in TBS, immune positive cells were detected using 3, 3' diaminobenzidine tetrahydrochloride (DAB) solution (3 mg/ml in Tris-HCl, pH 7,6 with 3 % H_2O_2). Sections were counterstained with Mayer's hematoxylin. The sections were observed on an Olympus BX51 microscope and images were captured using Olympus DP70 camera with DP controller software (Ver. 3.1.1.267). No immune-positivity were detected in both blocking control (**a**) and negative tissue (trachea) control (**b**) sections. Scale bars are 50 µm.
